# Acute generalised exanthematous pustulosis associated with upadacitinib treatment

**DOI:** 10.1002/ski2.444

**Published:** 2024-08-15

**Authors:** Thandiwe Banda, Sanaa Butt, Madhavi Maheshwari, Moumita Chattopadhyay

**Affiliations:** ^1^ Dermatology Department Birmingham City Hospital Sandwell and West Birmingham Hospital Trust Birmingham UK; ^2^ Histopathology Department Royal Wolverhampton NHS Trust Wolverhampton UK

## Abstract

Acute generalised exanthematous pustulosis (AGEP) is a rare drug‐induced pustular eruption characterised by the rapid onset of superficial pinhead pustules. We discuss the case of a 27‐year‐old man who presented with a generalised pustular eruption on the neck, trunk and limbs. He commenced upadacitinib for the treatment of atopic dermatitis (AD) 6 months before developing the rash, and the dose was increased from 15 to 30 mg daily, 3 months prior. His only other medication was oral terbinafine, for suspected tinea corporis, which was initiated 1 month before developing the pustular eruption. Laboratory investigations showed a mildly raised CRP 25 mg/L, neutrophilia 8.22 10 × 9/L, and a mildly raised ALT 46 U/L. A skin biopsy showed subcorneal pustules and a few scattered keratinocytes. Upadacitinib and terbinafine were suspended and the pustular eruption resolved. Updacitinib was reintroduced 3 weeks later as the rash was thought to be due to terbinafine and the rash recurred. He was diagnosed with AGEP secondary to upadacitinib. Upadacitinib is a selective JAK inhibitor that is increasingly used for the management of AD and clinicians should be aware that AGEP is a rare but severe adverse effect.

## Graphical abstract

A 27‐year‐old man presented to the dermatology department with a pustular eruption following treatment with upadacitinib for atopic dermatitis. The pustular eruption resolved after suspension of the drug and he was diagnosed with acute generalised exanthematous pustulosis secondary to upadacitinib.

## 1 CASE REPORT

**FIGURE 1:**
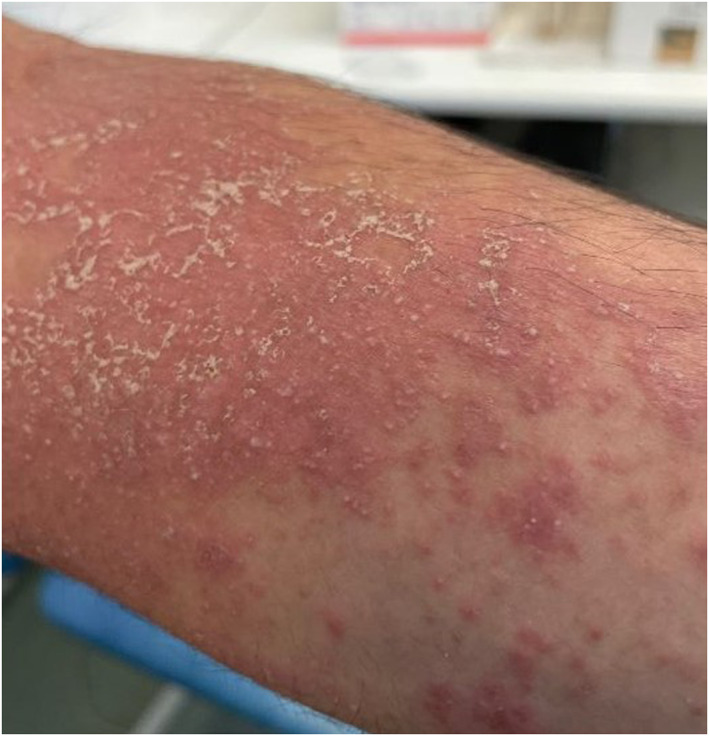
Pustular eruption on left forearm 1 day after pustules developed.

**FIGURE 2:**
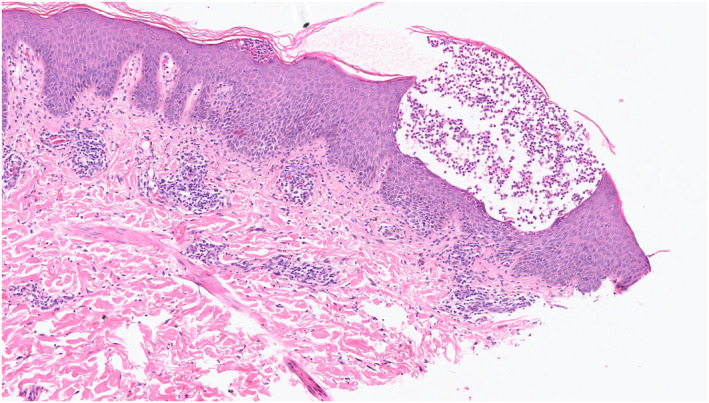
Histology showing a subcorneal pustule with a few necrotic keratinocytes. The epidermis underneath and adjacent to the pustules shows mild spongiosis with inflammatory cell exocytosis.

We present the case of a 27‐year‐old man who developed a pustular eruption following treatment with upadacitinib, a selective oral JAK1 inhibitor, for severe atopic dermatitis (AD). Upadacitinib was commenced at 15 mg daily following inefficacious treatment with phototherapy and then ciclosporin. Three months after initiation, the dose was increased to 30 mg daily due to limited efficacy. One month following dose increase, he developed mostly circular and few annular, erythematous, scaly patches around his neck, trunk and limbs (Figure 1). A month's course of oral terbinafine was completed for suspected tinea corporis yet the rash continued to worsen and he developed pustules. Clinical examination revealed facial erythema, mild periorbital oedema and generalised pustular rash on an erythematous base on lateral neck, posterior auricular areas, flexural sites, chest and the right forearm. He had no oral, genital or eye involvement. Laboratory investigations showed a mildly raised CRP 25 mg/L (normal value < 3), neutrophilia 8.22 10*9 L (normal value < 7.5), eosinophilia 0.57 10 × 9/L (normal value < 0.5) and mildly raised ALT 46 U/L (normal value < 41). Differentials included acute generalised exanthematous pustulosis (AGEP), generalizsed pustular psoriasis (GPP) and symmetrical drug‐related intertriginous and flexural exanthema (SDRIFE). Skin biopsy showed several subcorneal pustules of various sizes with few scattered necrotic keratinocytes. No microorganisms were detected within the pustules on histology (Figure 2). The papillary dermis was minimally oedematous and showed mixed inflammatory cell infiltrates of polymorphs, lymphocytes and occasional eosinophils. Terbinafine and upadacitinib were suspended and he was treated as an inpatient with IV flucloxacillin and emollients, following which the rash improved and entirely resolved. At 3 weeks follow‐up, the patient's eczema returned and upadacitinib was restarted at 30 mg daily as Terbinafine was thought to be the trigger. The pustular rash reappeared within 2 days of restarting upadacitinib and resolved 3 days after cessation.

We suspect this was a case of AGEP secondary to upadacitinib based on the clinical features of flexural distribution, onset following initiation of the higher dose of upadacitinib, recurrence after rechallenge, rapid resolution following drug cessation, neutrophilia and eosinophilia detected in blood tests and histological features (Table 1). Upadacitinib is increasingly used for AD due to its more desirable side‐effect profile compared to other JAK inhibitors because of its selectivity for the JAK1 receptor.^2^ There is limited literature with regard to pustular eruptions secondary to JAK inhibitors but two cases of palmoplantar pustulosis have been reported in association with baricitinib and tofacitinib.^3^, ^4^ AGEP is triggered due to the stimulation of drug‐specific T cells which produce cytotoxic proteins that cause keratinocyte apoptosis with formation of subcorneal vesicles. In addition, various chemokines cause neutrophil chemotaxis leading to formation of pustules.^1^ The pathophysiology by which upadacitinib triggers AGEP remains unknown; however, it is possible that the IL36RN gene mutation could play a role by leading to an increased production of inflammatory cytokines IL‐6, IL8, IL1ɑ and IL1β, therefore increasing susceptibility to AGEP.^5^ We highlight this case to raise awareness of this rare but significant adverse reaction to upadacitinib as it becomes more widely used for the treatment of AD.

**TABLE 1:** Factors favouring the diagnosis of AGEP over pustular psoriasis.^1^

	AGEP	GPP
History of psoriasis (family/personal)	Usually lacking	Often present
Distribution pattern	Initially predominance in the folds	More generalised
Onset of pustules	Fast (hours or few days after use of medication)	Slower
Duration of pustules	Shorter (rapid resolution in a few days, max. 15 days, after drug suspension)	Longer
Size of pustules	Tiny (pinhead)	Larger
Duration of eruption/fever	Shorter (resolution in a few days after drug suspension)	Longer
History of drug reaction	Usual	Uncommon
Recent drug administration	Very frequent	Less frequent
Arthritis	Rare	About 30%
Histology	Single‐cell necrosis of keratinocytes, oedema of papillary dermis, vasculitis and exocytosis of eosinophils	Papillomatosis, acanthosis, tortuous or dilated vessels

Abbreviations: AGEP, acute generalised exanthematous pustulosis; GPP, generalised pustular psoriasis.

## CONFLICT OF INTEREST STATEMENT

None to declare.

## AUTHOR CONTRIBUTIONS

**Thandiwe Banda**: Writing – original draft (lead). **Sanaa Butt**: Writing – original draft (supporting). **Madhavi Maheshwari**: Writing – review & editing (equal). **Moumita Chattopadhyay**: Writing – review & editing (equal).

## ETHICS STATEMENT

Not applicable.

## PATIENT CONSENT

Patient has consented to images being used in open publication.

## Data Availability

Data sharing is not applicable to this article as no new data were created or analysed in this study.
